# Single-cell and spatial multi-omics for studying cell competition in the tumor microenvironment

**DOI:** 10.3389/fonc.2026.1971798

**Published:** 2026-10-30

**Authors:** Prakash Balu, Ashna Sureshkumar, Gayathri Devi Sundaramurthy, Geetha Subramaniam, Ling Shing Wong

**Affiliations:** 1 Department of Biotechnology, Vels Institute of Science, Technology and Advanced Studies, Chennai, Tamil Nadu, India; 2 Faculty of Health and Life Sciences, INTI International University, Nilai, Negeri Sembilan, Malaysia; 3 University of Virginia, Charlottesville, VA, United States; 4 Department of Biochemistry, Biotechnology and Bioinformatics, Avinashilingam Institute for Home Science and Higher Education for Women, Coimbatore, Tamil Nadu, India

**Keywords:** artificial intelligence, cancer, cell competition, hypothesis generation, multi-omics integration, single-cell RNA sequencing, spatial proteomics, spatial transcriptomics

## Abstract

Cell competition is a conserved fitness-surveillance process, first described in Drosophila epithelia, by which cells compare relative fitness with their neighbors so that less-fit “loser” cells are eliminated, extruded or forced to differentiate while fitter “winner” cells expand. In the tumor microenvironment (TME), this logic appears to be co-opted by transformed clones that eliminate normal epithelium, outcompete neighboring subclones and reshape stromal and immune compartments, making cell competition a potentially underappreciated contributor to cancer clonal evolution, immune escape and therapy resistance. Because competition is defined by a comparison between adjacent cells and by a demonstrated change in the relative representation of one population, it is difficult to resolve with bulk sequencing, which averages across precisely the heterogeneity that defines it. The convergence of single-cell RNA sequencing (scRNA-seq), spatial transcriptomics, spatial and single-cell proteomics, artificial intelligence (AI) and network-inference bioinformatics now allows competition-relevant cellular states, interfaces and signaling relationships to be measured at the resolution the problem requires. We emphasize at the outset that these technologies are, in almost all current applications, hypothesis-generating: they identify candidate competitive interfaces and putative winner and loser states from static measurements, and they do not by themselves demonstrate that competition has occurred. This review synthesizes advances across these domains; proposes an explicit three-tier evidence framework separating direct experimental evidence of cell competition from competition-relevant associations and from computational predictions; distinguishes cell competition from related processes such as clonal selection, immune editing and differential proliferation; and defines a putative cell competition network as a falsifiable construct whose edges include ligand-receptor, spatial-adjacency, metabolic and mechanical relations. We further outline a conceptual, context-dependent Cell Competition Score (CCS) as a testable specification rather than a biomarker-ready instrument, and set out the benchmarking, causal-inference and prospective-validation requirements that stand between current capability and any clinical application.

## 1 Introduction

Tumors are not homogeneous masses of malignant cells but ecosystems in which cancer cells, fibroblasts, endothelial cells and diverse immune populations coexist and compete for space, nutrients and survival signals. Cell competition is the process by which cells compare relative fitness with their neighbors, so that less-fit “loser” cells are actively eliminated by apoptosis, extrusion or forced differentiation while fitter “winner” cells proliferate to fill the vacated space (1). The phenomenon was first characterized in Drosophila imaginal discs, where cells heterozygous for Minute mutations in ribosomal protein genes were outcompeted by wild-type neighbors despite being perfectly viable in isolation, establishing that fitness is a relative, context-dependent property rather than an absolute one (2).

In mammalian tissues, cell competition functions as a quality-control mechanism. Epithelial defense against cancer (EDAC) extrudes newly transformed cells before they can establish a tumor, and fitness sensors including p53, MYC and the Hippo-YAP pathway police cell populations during development and homeostasis (3, 4). Cancer appears to subvert this same surveillance logic. Once a clone acquires a sufficient fitness advantage — through elevated MYC activity, p53 pathway alteration, altered ribosome biogenesis or mechanical stiffening — it can behave as a “super-competitor” capable of eliminating both normal epithelium and less-fit neighboring subclones, a behavior increasingly implicated in clonal selection, intratumoral heterogeneity and metastatic dissemination (5–8).

The consequences extend beyond tumor initiation. Competition has been described in primary and metastatic disease (9), and competition-driven interactions between disseminated tumor cells and the surrounding tissue have been linked to metastatic latency and relapse (10). Mechanical determinants — cortical tension, crowding, interfacial contractility — are now recognized as being as decisive as biochemical ones in setting the direction of elimination (11).

Historically, cell competition has been studied using genetically engineered model organisms and two-dimensional culture, approaches that deliberately isolate the phenomenon from the complexity of a human tumor but that have the decisive advantage of allowing genetic labelling, longitudinal imaging and perturbation — the three things that together constitute direct evidence of competition. The TME, by contrast, is a multicellular, spatially structured and dynamically evolving system in which competitive interactions are shaped not only by cell-intrinsic fitness but by stromal architecture, immune infiltration, hypoxia and mechanical stress (5). Bulk genomic and transcriptomic profiling averages over this heterogeneity and cannot resolve which cells are winning, which are losing, or where these events occur within the tissue.

Over the past five years, single-cell and spatial omics technologies have matured to the point where the cellular states and tissue configurations associated with competition can be measured at the required resolution in human material. scRNA-seq resolves the transcriptional states of candidate winner and loser populations; spatial transcriptomics preserves the tissue coordinates needed to identify where candidate competitive interfaces lie; spatial and single-cell proteomics add the protein- and post-translational-level resolution that better reflects functional cell state; and AI, particularly single-cell foundation models and network-inference algorithms, provides computational scaffolding to integrate these layers. It is important to be explicit about what this does and does not achieve. These are static, largely cross-sectional measurements of human tissue. They can establish that two populations with divergent fitness-associated states are in contact, and they can nominate the signaling and metabolic relationships that might mediate an interaction between them; they cannot, on their own, establish directionality or demonstrate that one population is eliminating or displacing the other. Throughout this review we therefore describe the object these methods produce as a putative, or inferred, cell competition network, and reserve the unqualified term “cell competition” for situations in which the criteria set out in Section 2 have been met.

This review synthesizes progress in each domain, proposes an explicit evidence framework and a falsifiable network formalism, and considers what would be required before competition-network features could be considered for translational use.

### 1.1 Scope and literature-selection strategy

This is a narrative review, not a systematic or scoping review, and it does not claim exhaustive coverage of the primary literature. To make the basis of selection transparent, we state our approach explicitly. We searched PubMed/MEDLINE, Scopus and Google Scholar between January 2024 and June 2026 using combinations of the terms “cell competition”, “super-competitor”, “winner cell”, “loser cell”, “cell extrusion”, “epithelial defense against cancer”, “tumor microenvironment”, “single-cell RNA sequencing”, “spatial transcriptomics”, “spatial proteomics”, “cell-cell communication inference”, “ligand-receptor”, “foundation model” and “benchmarking”. bioRxiv and eLife reviewed preprints were searched separately and are treated as a distinct evidence class (see below).

Because the field spans developmental biology, biophysics, cancer genomics and computational methodology, no single date range was appropriate. For the mechanistic biology of cell competition we prioritized primary studies of any date that established a mechanism with genetic or pharmacological perturbation, together with recent authoritative reviews. For technology and computational methodology we restricted attention largely to 2020-2026, since platform capabilities and benchmarking evidence before that period are no longer representative. Studies were prioritized when they (i) reported a defined molecular or mechanical mechanism together with a measured competitive outcome, (ii) were performed in mammalian systems or human tissue, or (iii) provided systematic benchmarking of a computational method. We excluded studies in which the term “competition” was used metaphorically, without a measured change in the relative representation of one population. Two limitations follow directly from this design: the selection of illustrative examples reflects the authors’ judgment rather than a pre-registered protocol, and the balance of examples is weighted towards the well-studied tumor types for which single-cell and spatial atlases exist.

Preprints are cited in only two places in this review (references 18 and 28). Both are explicitly labeled as preprints in the reference list and in the text, neither supports a load-bearing mechanistic or translational claim, and the surrounding language is hedged accordingly. Should the journal require review articles to be based exclusively on peer-reviewed work, both citations and the sentences they support can be removed without affecting the argument of the review; the corresponding statements are also supported by the peer-reviewed references cited alongside them (10, 11, 16, 17).

## 2 What counts as evidence of cell competition?

Much of the difficulty in applying single-cell and spatial technologies to cell competition is definitional rather than technical. Cell competition is not a cellular state, a spatial pattern or a signaling relationship; it is an outcome — a context-dependent change in the relative representation of one population brought about by the presence of another. Demonstrating it therefore requires more than demonstrating that the conditions permitting it are present.

We propose that four criteria must be satisfied before an observation is described as cell competition. First, relative fitness difference: two genetically or phenotypically distinguishable populations must differ in a fitness-associated property. Second, physical proximity or direct contact: the two populations must interact within the range over which the proposed mechanism can operate, whether by contact, by short-range diffusible signal or by competition for a locally limiting resource. Third, directionality: the interaction must be asymmetric, with one population identified as the winner and the other as the loser. Fourth, and most demanding, outcome: the less-fit population must be shown to undergo elimination, extrusion, forced differentiation or displacement as a consequence of the interaction, and this must be shown to be context-dependent — that is, the loser population must be viable when the winner is absent. This last requirement is what distinguishes competition from intrinsic cell death or from simple differences in proliferation rate, and it is almost never testable in a single fixed human tissue section.

It follows those observations in human tumor material occupy different evidentiary tiers, which we define in **Table 1** and use consistently throughout this review. Tier 1 comprises direct experimental evidence, in which co-culture, genetic labelling, longitudinal imaging or lineage tracing demonstrates a context-dependent competitive outcome, ideally with a perturbation that reverses or abolishes it. Tier 2 comprises competition-relevant associations: measurements establishing that fitness-divergent populations are adjacent, that boundary cells display stress or apoptotic-priming programs, or that a neighborhood configuration is associated with outcome. Tier 3 comprises computational predictions, including inferred ligand-receptor edges, foundation-model embeddings and in silico perturbation responses. Tier 2 and Tier 3 observations generate candidate competitive interactions; they do not establish them. Almost all of the human-tissue examples discussed in Sections 4 to 7 of this review are Tier 2 or Tier 3, and we flag them as such.

**Table 1:** A three-tier evidence framework for claims about cell competition. Tiers 2 and 3 identify candidate interactions requiring functional validation; only Tier 1 demonstrates competition.

Evidence tier	What is demonstrated	Typical approaches	Permissible claim
Tier 1 Direct experimental evidence	A context-dependent competitive outcome: the loser population is eliminated, extruded, forced to differentiate or displaced in the presence of the winner, and is viable in its absence. Directionality established; ideally reversed by perturbation.	Mosaic co-culture; genetic mosaic models; clonal labelling and lineage tracing; longitudinal live imaging; organoid mixing assays with perturbation	“Cell competition occurs”; a named mechanism may be assigned
Tier 2 Competition-relevant association	Fitness-divergent populations are in contact; boundary cells show stress, apoptotic-priming or extrusion markers; neighborhood configuration associates with clonal or clinical outcome. Directionality and outcome not established.	Single-cell-resolution spatial transcriptomics and proteomics; neighborhood analysis; interface-anchored differential expression; paired multimodal profiling	“A tissue configuration consistent with competition”; “candidate competitive interface”
Tier 3 Computational prediction	A statistical or model-derived relationship between populations, derived from co-expression, embedding geometry or learned priors. No physical or causal claim is established by the method itself.	Ligand-receptor inference; spatial-domain models; foundation-model embeddings; in silico perturbation prediction	“Putative”, “predicted”, “hypothesis-generating”; requires orthogonal validation

### 2.1 Distinguishing cell competition from related processes

Several processes discussed in this review overlap with cell competition without being equivalent to it, and conflating them is the most common error in the applied literature. Clonal selection describes a change in clone frequency that may arise from cell-autonomous fitness differences alone, with no interaction between clones; competition is a specific, interaction-dependent subset of it. Immune editing involves elimination of tumor cells by cytotoxic lymphocytes through antigen recognition, a mechanistically distinct process, although the MHC class I-LILRB3 axis discussed below shows that the boundary is genuinely blurred in some settings. Differential proliferation produces changing population ratios without any elimination of the slower population. Therapy-induced plasticity produces transcriptional-state changes that can mimic winner-to-loser conversion in cross-sectional data. Nutrient limitation causes cell death when severe, whether or not a fitter neighbor is present; it constitutes competition only when the fitter neighbor is shown to cause the deprivation or its consequences. Generic cell-cell communication is ubiquitous and is competition only when it produces an asymmetric fitness outcome. **Table 2** summarizes these distinctions and the additional evidence required in each case.

**Table 2:** Cell competition and related processes: overlapping phenomena, distinguishing features and the additional evidence required before a competitive interpretation is warranted.

Process	How it resembles cell competition	How it differs	Additional evidence required
Clonal selection	Relative clone frequencies change over time; fitter clones expand	May be entirely cell-autonomous; does not require interaction between clones or elimination of the minority clone	Show that the outcome depends on the presence of the other clone (context dependence)
Immune editing	Less-fit or antigenic cells are eliminated by neighboring cells	Elimination is antigen-driven and executed by professional immune effectors, not by fitness comparison at an epithelial interface	Antigen-independence, or demonstration of a fitness-sensing mechanism such as Flower or MHC-I-LILRB3
Differential proliferation	One population comes to dominate	No elimination, extrusion or forced differentiation of the other population	Direct measurement of loser death, extrusion or displacement, not only of ratio change
Therapy-induced plasticity	Cells shift between fitness-associated transcriptional states	A state change within one population rather than an interaction between two	Lineage tracing distinguishing state transition from selective elimination
Nutrient or hypoxic limitation	Cells die where a resource is scarce; stress programs are activated	Death occurs whether or not a fitter neighbor is present	Show that the neighbor causes the deprivation or its downstream consequence (e.g., ref. 12)
Generic cell-cell communication	Ligand-receptor signaling between adjacent populations	Signaling is ubiquitous, frequently reciprocal, and usually not associated with any fitness outcome	Asymmetric edge weight plus a measured fitness consequence in the receiving population
Immune exclusion/spatial architecture	Structured neighborhoods separate populations and track with outcome	Describes tissue organization, not a demonstrated winner-loser interaction	Longitudinal sampling showing that one population displaces the other

## 3 Fundamentals of cell competition in the tumor microenvironment

Cell competition in cancer operates through at least three overlapping mechanistic modes: competition for trophic or survival factors, direct fitness sensing through cell-surface mismatch, and mechanical or contact-dependent competition (4, 9). These modes are summarized in **Table 3** and illustrated schematically in **Figure 1**. All of the studies cited in this section constitute Tier 1 evidence in the framework of **Table 1**; they were established in tractable experimental systems in which perturbation and longitudinal observation were possible, and it is precisely this that the human-tissue studies discussed later cannot yet provide.

**Table 3:** Mechanistic modes of cell competition and their molecular determinants, with the experimental system in which each was established.

Mode	Fitness signal/sensor	Key molecular players	Competitive outcome	Model system	Primary reference
Metabolic/factor-limited	Extracellular L-proline availability read out by the ISR	GCN2-eIF2a-ATF4; proline transporters	Winners induce increased proline uptake in losers, repressing the cytoprotective ISR and driving loser apoptosis	Mouse embryonic stem cells; early embryo	Krishnan et al., 2025 (12)
Trophic competition with myeloid cells	Engulfment-dependent mTORC1 signaling under dietary protein restriction	MYC, mTORC1, TFEB/TFE3, PIKfyve	Reprogramed macrophages outcompete MYC-high tumor cells; tumor growth slows	Mouse breast cancer, low-protein diet	Zhang et al., 2023 (13)
Fitness fingerprint: Flower code	Mismatched Flower isoforms at clone boundaries	Flower (Win/Lose isoforms), Azot	Less-fit neighbor is marked and cleared by caspase-dependent apoptosis	Drosophila; mammalian conservation of this module not fully established	Merino et al., 2015 (14)
Fitness fingerprint: innate-immune surface recognition (mechanistically distinct from the Flower code)	MHC class I on transformed cells engaging LILRB3 on normal epithelium	MHC-I, LILRB3, SHP2-ROCK	Wild-type cells generate contractile force that extrudes the transformed cell	MDCK and human keratinocyte models	Ayukawa et al., 2021 (15)
Oncogene dosage (super-competition)	Relative, not absolute, MYC level between adjacent clones	MYC and MYC target program	Higher-MYC clone expands; lower-MYC clones undergo extinction	Genetic barcoding in mouse glioma with mathematical modeling	Ceresa et al., 2023 (7)
Tumor-suppressor checkpoint	Relative p53 pathway activity (direction is context-dependent; compare mechanical mode below)	p53; PUMA/NOXA	p53-mutant cells behave as winners; neighbors eliminated or forced to differentiate	Pluripotent stem cells; epithelium	Perez Montero et al., 2024 (20)
Mechanical/density-dependent	Differential sensitivity to compaction and crowding	p53, YAP/TAZ-TEAD, E-cadherin, actomyosin	Crowding-sensitive or more compressible cells are compacted and live-extruded	MDCK monolayers; Drosophila notum; epithelial explants	Wagstaff et al., 2016 (16); Levayer et al., 2016 (17); Gupta et al., 2025 (18) [preprint]
Epithelial defense against cancer (EDAC)	Recognition of a newly transformed cell by surrounding wild-type epithelium	COX-2/PGE2 axis; modulated by obesity	Apical extrusion of the transformed cell before tumor initiation	MDCK; mouse intestinal and pancreatic epithelium	Sasaki et al., 2018 (25); Sato et al., 2020 (26)
Niche-level clonal competition	Secreted-factor suppression of neighboring stem cells	APC, NOTUM, WNT	Apc-mutant supercompetitors silence and displace wild-type crypt stem cells	Mouse intestinal crypt	van Neerven et al., 2021 (23); Flanagan et al., 2021 (24)
Competition-associated phenotypic plasticity (provisional)	Overcrowding at high local density in the primary tumor	Adherent-to-suspension transition factors	Displaced losers reported to be reprogramed towards a CTC-like suspension state rather than dying	Disseminated tumor cell models	Huh et al., 2024 (28) [preprint]

All entries represent Tier 1 evidence obtained in experimental systems, with the exception of the final row, which rests on a preprint.

**Figure 1:**
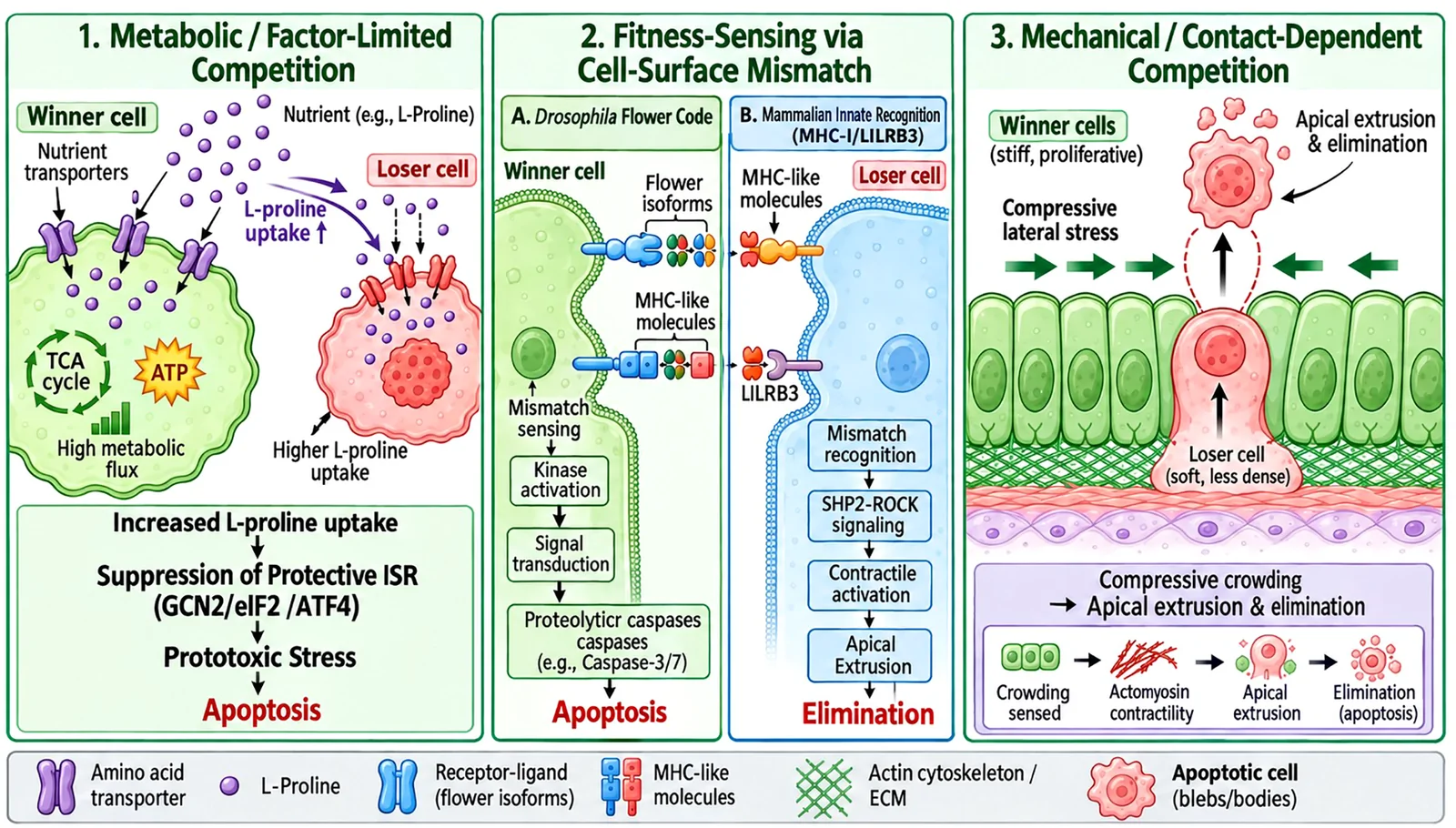
Three mechanistically distinct modes of winner-loser cell competition.

In factor-limited competition, the availability of a limiting metabolite determines outcome. Sensing of extracellular L-proline by the integrated stress response (ISR) has been shown to determine competitive outcome in the early mouse embryo and in mouse embryonic stem cell co-cultures. Loser cells in this system have mitochondrial dysfunction, which causes amino-acid deprivation and consequent ISR activation; importantly, the ISR is cytoprotective in these cells and is required for their survival. In a competitive environment, winner cells induce increased L-proline uptake in the loser cells, and it is this increase — not deprivation — that represses the protective ISR and precipitates loser elimination (12). The direction of this mechanism is counterintuitive and is easily misdrawn: elimination follows proline sufficiency in the loser, not proline starvation. The authors accordingly interpret competition in this setting as a nutrient sensor that spares dysfunctional cells when amino acids are scarce and removes them when they are plentiful. A complementary *in vivo* demonstration comes from the myeloid compartment: tumor-associated macrophages can be dietarily or genetically reprogramed, via engulfment-dependent mTORC1 signaling, to outcompete MYC-overexpressing cancer cells and slow tumor growth (13). This is among the clearest evidence that competitive balance is a therapeutically addressable variable rather than a fixed property of a tumor.

In fitness-sensing competition, cells compare surface molecules at clone boundaries. Two distinct mechanisms are frequently grouped together and should not be. In Drosophila, differential display of Flower isoforms, together with the downstream factor Azot, marks the less-fit population for caspase-dependent elimination and maintains tissue health and lifespan (14); conservation of this specific module in mammals remains incompletely resolved. Separately, and through entirely different molecules, an innate-immune recognition mechanism operates in mammalian epithelia: MHC class I on RasV12-transformed cells engages LILRB3 on surrounding wild-type epithelial cells, triggering SHP2-ROCK-dependent contractile activation that extrudes the transformed cell (15). These are two independent fitness-fingerprint systems, not two illustrations of one pathway. What they share is only the logic — a surface-level comparison at a clone boundary — and the observation that surface sensing and mechanical elimination are often the same event viewed at different scales.

In mechanical competition, differences in sensitivity to crowding and compaction determine outcome. Cells with relatively high p53 activity become hypersensitive to crowding-induced compaction and are eliminated by neighbors with lower p53 activity (16), while tissue crowding induces caspase-dependent competition for space in the Drosophila notum (17). Recent biophysical work reports that relative compressibility is a defining loser characteristic and that mechanical imbalance between normal and transformed cells contributes to epithelial homeostasis through competition; this work is currently available as a reviewed preprint and should be treated as provisional (18). The direction of elimination — apical or basal, within or out of plane — is set by the nature of the forces at the interface, with direct consequences for whether a displaced cell is cleared or released into the stroma (11).

A recurring set of transcription factors and pathways underlies fitness sensing across these modes, but none of them specifies winner or loser status independently of context. MYC dosage differences alone are sufficient to trigger elimination of lower-MYC cells in the early mammalian embryo (19), and analogous MYC-driven super-competition shapes clonal selection during early gliomagenesis, where barcoding revealed a large early clonal extinction event whose magnitude correlated with MYC target activity (7, 8). p53 functions as a fitness checkpoint, and p53 mutation increases the competitive ability of pluripotent stem cells (20) — yet in the mechanical setting of reference 16 it is high p53 activity that marks the loser, so the sign of the p53 term depends on the mode of competition in operation. The Hippo-YAP pathway is equally context-dependent: peritumoral activation of YAP and TAZ suppresses liver cancer in mice by allowing normal hepatocytes to outcompete transformed cells (21), while high YAP activity confers crowding resistance and winner status on genetically variant human pluripotent stem cells (22). Whether MYC, p53 or YAP/TEAD activity marks a winner or a loser is therefore a property of the tissue and the competitive mode, not of the pathway. This is the single most important caveat for any signature-based assignment of competitive status, and it recurs in our treatment of the Cell Competition Score in Section 9.

Beyond tumor-intrinsic clonal competition, the same logic structures the surrounding tissue. Apc-mutant cells act as supercompetitors in intestinal tumor initiation, in part by secreting NOTUM to bias clonal competition against wild-type crypt stem cells (23, 24). Conversely, EDAC eliminates transformed cells before tumor initiation, and this protection is attenuated by obesity and by COX-2/PGE2 signaling, linking systemic and inflammatory state to the competitive threshold for tumor initiation (25, 26). At the level of whole tissues, deep sequencing of morphologically normal human bronchial epithelium shows extensive mutant clonal colonization (27); we note that this observation is consistent with competitive constraint but does not by itself demonstrate it, since clone-size distributions of this kind can also be generated by neutral drift and cell-autonomous fitness differences.

Competitive elimination may not always be terminal. It has been reported, currently in preprint form only, that under high-density conditions displaced loser cells can be reprogramed towards an adherent-to-suspension state resembling circulating tumor cells rather than simply dying (28). We cite this as a provisional hypothesis rather than as established biology; it is, however, broadly consistent with the peer-reviewed observation that competitive interactions shape metastatic latency and relapse (10). Collectively, these findings establish cell competition as a multi-scale process spanning subcellular signaling, single-cell fate decisions and tissue-level architecture — a process whose components single-cell and spatial multi-omic technologies are now positioned to measure, if not yet to demonstrate, in human tissue.

## 4 Single-cell RNA sequencing: resolving candidate winner and loser transcriptional states

scRNA-seq is the primary workhorse for dissecting cellular heterogeneity within the TME, enabling unbiased identification of malignant subclones, stromal populations and immune states across essentially every major cancer type. Because it captures the transcriptomes of thousands to millions of individual cells simultaneously, it is well suited to separating the transcriptional programs of candidate winner and loser populations that would otherwise be averaged away in bulk RNA-seq — including rare subclones, transitional states, and the stress-response and apoptotic-priming programs associated with elimination (4, 12). It is worth stating plainly what this does not do: a transcriptional state consistent with competitive potential is not evidence that competition has occurred, and dissociated data contain no information about which cells were adjacent.

The mechanistic literature supplies the signatures to look for. Loser cells in the early mouse embryo display a characteristic profile of low mTOR signaling, elevated p53 activity, low MYC and reduced expression of protein-synthesis genes, together with impaired mitochondrial function (12, 19). Winner status in mechanically competitive settings is associated with high YAP/TEAD target activity and crowding resistance (22). These are testable transcriptional definitions rather than *post hoc* labels, and they make a competition-oriented reanalysis of existing tumor atlases feasible without new tissue collection. They are also, as Section 3 emphasized, tissue- and mode-specific; transporting them into a new context without revalidation is not justified.

Applied to tumors, single-cell profiling has surfaced competition-relevant biology. Barcoded lineage tracing combined with bulk and single-cell transcriptomics in gliomagenesis showed that MYC target activity correlated more strongly with clonal size imbalance than any other measured program, and that experimental MYC downregulation was sufficient to convert a clone into a loser (7) — the lineage tracing and the perturbation, rather than the transcriptomics, are what make this Tier 1 evidence. In metastatic melanoma, longitudinal CITE-seq identified heterogeneous tumor subclones, rare gamma-delta T cells and plasmacytoid dendritic cells, and resolved patient-specific cellular communities associated with innate resistance, acquired resistance or durable response to immune checkpoint blockade (29). That study is a Tier 2 observation with respect to competition: it describes microenvironmental organization associated with clinical outcome and does not test a winner-loser relationship. Study designs of this kind — longitudinal, treatment-paired and multimodal — are nevertheless the natural substrate for competition analysis, because competitive outcome is a change over time rather than a state at one moment.

scRNA-seq alone cannot resolve the spatial context in which candidate competitive interactions occur, nor can it capture protein-level or post-translational determinants of fitness. Both are essential limitations, because cell competition is by definition a contact- and neighbor-dependent phenomenon: dissociation destroys precisely the adjacency information that distinguishes a candidate competitive interaction from coincidental co-occurrence of two cell states in the same tumor. This motivates integration with the spatial and proteomic technologies discussed in the following sections and summarized as an end-to-end workflow in **Figure 2**.

**Figure 2:**
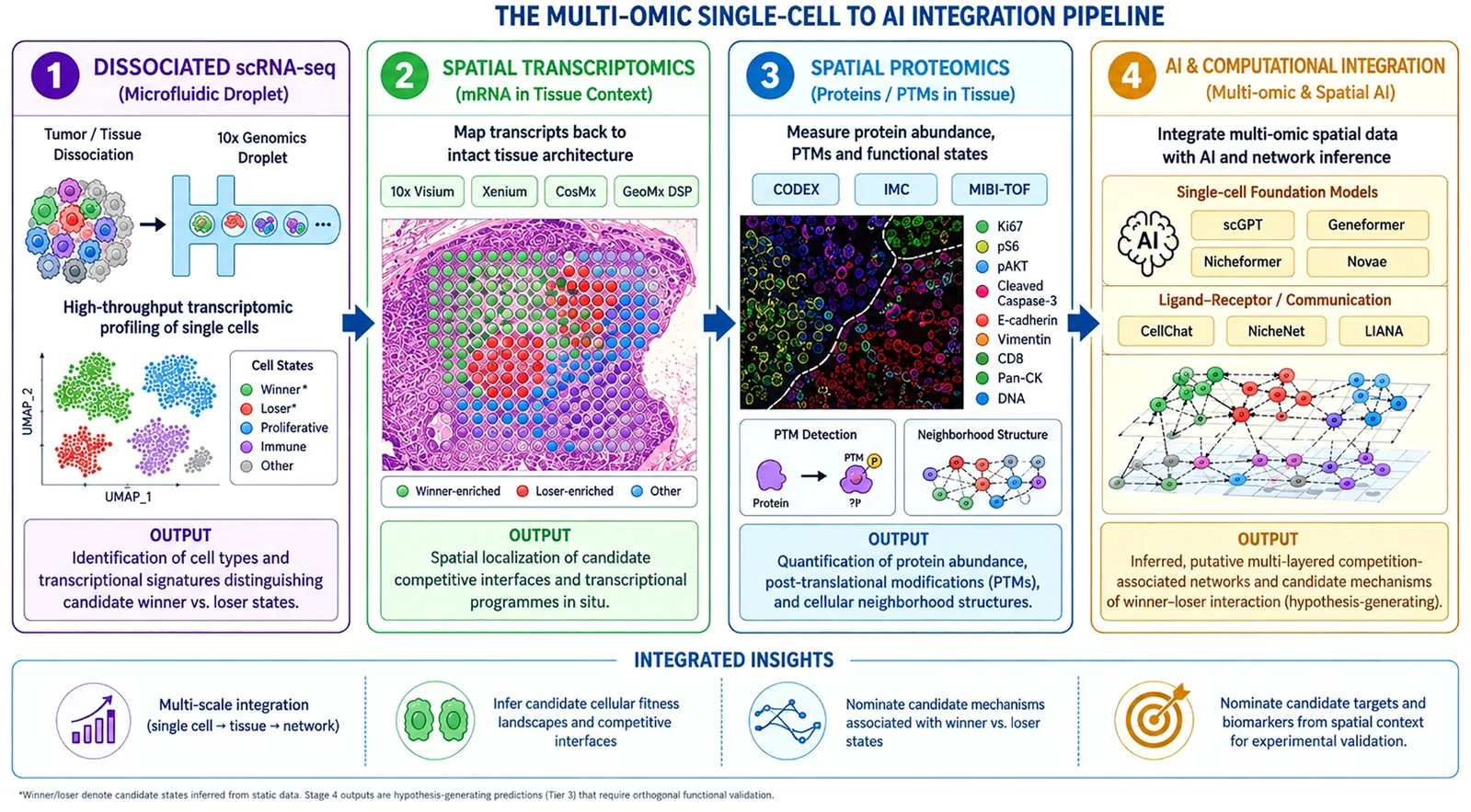
The multi-omic single-cell to computational analysis pipeline.

## 5 Spatial transcriptomics: localizing candidate competitive interfaces in situ

Because cell competition is defined by comparison between neighboring cells, spatial context is not an optional refinement but a mechanistic requirement. Spatial transcriptomics preserves the tissue coordinates of gene expression measurements, complementing the cellular resolution of scRNA-seq with the positional information needed to identify candidate competitive interfaces, clonal boundaries and tumor-stroma-immune niches. Current platforms span a wide range of resolutions and coverage, and the trade-offs between them map directly onto what can and cannot be claimed (**Table 4**). Spatial proximity satisfies the second of the four criteria in Section 2; it does not satisfy the third or the fourth.

**Table 4:** Spatial transcriptomic platforms compared on the axes that determine whether candidate competitive interfaces can be resolved.

Platform	Spatial resolution	Transcript coverage	Contribution to competition analysis	Principal limitation
Spot-based array capture (e.g. Visium)	Multi-cell spots (~55 um)	Transcriptome-wide, untargeted	Unbiased discovery of regional fitness signatures; first-pass identification of candidate competitive zones	Spots average across winner and loser cells, destroying neighbor-level contrast; requires deconvolution
Continuous array with fine binning (e.g. Visium HD)	Near-single-cell binning	Transcriptome-wide, untargeted	Retains untargeted coverage while approaching the resolution needed to assign candidate winner or loser identity	Binning is not true segmentation; boundary cells remain ambiguous
*In situ* hybridization with cyclic imaging (e.g., Xenium)	Subcellular; single-cell segmentation	Targeted panel (hundreds of genes)	Visualization of individual candidate winner and loser cells with matched morphology; supports adjacency analysis	Panel must be pre-specified, so fitness markers must be hypothesized in advance; segmentation errors in dense tumor nests
Cyclic *in situ* imaging (e.g. CosMx SMI)	Subcellular; single-cell segmentation	Targeted panel; whole-transcriptome panels emerging	Single-cell-resolved maps with matched histology; suitable for defining candidate interfaces cell by cell	Same panel-design constraint; cost limits sample number and therefore cohort-level inference
Combinatorial barcoded smFISH (e.g. MERFISH)	Subcellular	Targeted, high-plex	High detection efficiency for quantitative comparison of fitness-pathway transcripts between adjacent clones	Demanding tissue quality and imaging time; limited archival FFPE applicability
Region-of-interest profiling (e.g. GeoMx DSP)	User-defined regions, not single cells	Transcriptome-scale RNA and protein panels	Designed to compare two adjacent but molecularly distinct regions — a natural fit for candidate winner-region versus loser-region contrasts	The comparison unit is the region, so the individual cell-cell contact event is not resolved
Bead or DNB array capture (e.g. Slide-seq, Stereo-seq)	~10 um to subcellular	Transcriptome-wide, untargeted	Combines untargeted coverage with fine resolution over large fields, useful for whole-section clone mapping	Lower per-cell transcript capture; segmentation must be inferred computationally

SMI, spatial molecular imager; FFPE, formalin-fixed paraffin-embedded; smFISH, single-molecule fluorescence *in situ* hybridization; DNB, DNA nanoball.

Sequencing-based, array-capture methods provide untargeted transcriptome-wide coverage but aggregate several cells per capture area, which destroys the neighbor-level contrast that defines competition and necessitates deconvolution. Imaging-based *in situ* platforms resolve individual transcripts within segmented single cells and preserve matched morphology, enabling candidate winner and loser cells to be visualized in their tissue context. The CosMx spatial molecular imager (SMI) achieves high-plex RNA and protein detection at subcellular resolution in formalin-fixed, paraffin-embedded (FFPE) tissue (30), and integrated single-cell, spatial and *in situ* analysis using Xenium has been used to resolve tumor subtypes, myoepithelial populations and boundary cells at the invasive front of human breast cancer (31). Boundary cells of that kind — expressing markers of two compartments simultaneously and located exactly at the interface — are the empirical objects a competition analysis needs, though they are equally consistent with transitional differentiation states and with segmentation error.

Region-of-interest profiling occupies a distinct niche. Because it is explicitly designed to compare two adjacent but molecularly distinct regions, it is a natural fit for candidate “winner region versus loser region” contrasts, at the cost of not resolving the individual cell-cell contact event. The choice between these designs should follow the question: interface-level mechanistic claims require single-cell segmentation, whereas cohort-scale associations between regional fitness signatures and outcome may not.

Segmentation is the rate-limiting step for all imaging-based approaches and deserves explicit attention in any competition study, because a segmentation error at a clone boundary manufactures exactly the artifact the analysis is trying to detect: apparent co-expression of winner and loser markers in one cell. Dedicated methods for cell segmentation in imaging-based spatial transcriptomics (32) and generalist segmentation algorithms (33) are now standard, but reported competitive interfaces should be accompanied by segmentation quality metrics rather than assumed correct.

A particularly informative class of application concerns cellular neighborhoods: spatial domains defined by local cell-type composition and organization rather than by expression clustering alone. Analysis of the colorectal cancer invasive front identified conserved cellular neighborhoods with distinct immune composition, including a granulocyte-associated neighborhood enriched for PD-1+CD4+ T cells whose presence was associated with improved patient survival (34). We note that this is an association between tissue architecture and outcome, and that the authors did not frame it as, or test it as, cell competition. It is relevant here for a narrower reason: it demonstrates that the neighborhood, rather than the whole section or the global cell-type frequency, is the appropriate spatial unit at which any competition-related score should be computed.

## 6 Spatial and single-cell proteomics: protein-level resolution of candidate competitive interactions

Cell competition is ultimately executed at the protein level — surface receptor mismatch, cytoskeletal tension, caspase activation, transporter engagement — so transcriptomic data alone give an incomplete picture. Every one of the mechanisms in **Table 3** terminates in a protein event that transcript abundance predicts only loosely. Spatial proteomics, named Nature Methods’ Method of the Year for 2024, has matured into a set of platforms capable of visualizing dozens of proteins simultaneously within intact tissue sections, capturing the functional and post-translational-modification (PTM) layer that transcriptomics cannot access (35, 36).

Several complementary platforms now exist, differing in chemistry, plex and throughput (**Table 5**). Metal-isotope antibody tags detected by time-of-flight mass spectrometry avoid the spectral overlap limitations of fluorescence and permit simultaneous profiling of tension, stress and apoptotic markers on the same section. Iterative fluorescent-oligonucleotide approaches achieve high plex on standard fluorescence microscopes at comparatively lower cost, which has helped broaden access to spatial proteomics across research and clinical laboratories. Cyclic immunofluorescence (CyCIF) offers a related iterative approach compatible with standard archival material, and combined transcriptomic-proteomic region profiling allows paired RNA and protein measurement from the same tissue regions (37).

**Table 5:** TOF-MS, time-of-flight mass spectrometry; MIBI-TOF, multiplexed ion beam imaging by time of flight; CODEX, co-detection by indexing; CyCIF, cyclic immunofluorescence; CyTOF, cytometry by time of flight.

Platform	Detection chemistry	Typical plex	Resolution	Competition-relevant readout	Principal constraint
Imaging mass cytometry	Metal-isotope antibody tags; laser ablation and TOF-MS	~40+ markers	Approx. 1 um, subcellular	No spectral overlap, so tension, stress and apoptotic markers can be co-measured on one section	Ablation is destructive; throughput and field of view limited
MIBI-TOF	Metal-isotope antibody tags; ion beam and TOF-MS	~40+ markers	Subcellular	High sensitivity for low-abundance fitness markers at clone boundaries	Limited instrument availability; slow acquisition
CODEX/PhenoCycler	Iterative hybridization and stripping of fluorescent oligonucleotide barcodes	~40-60+ markers	Single-cell, standard fluorescence microscopy	Established basis for cellular-neighborhood analysis, the spatial unit adopted here for defining candidate competitive niches	Cyclic imaging time; antibody-barcode conjugation validation burden
CyCIF	Iterative immunofluorescence with signal inactivation	~20–60 markers	Single-cell	Low-cost entry route; compatible with standard FFPE archives, enabling retrospective cohorts	Photobleaching and tissue degradation across cycles
Region-of-interest protein profiling	Photocleavable oligo-tagged antibodies, region collection	~100 targets	Region of interest	Paired RNA and protein from the same region, supporting multi-layer scoring	Not single-cell; interface events are averaged within the region
Suspension mass cytometry (CyTOF)	Metal-isotope antibody tags, suspension TOF-MS	~40–50 markers	Single cell, no spatial context	Deep phenotyping of dissociated candidate winner and loser populations; anchors spatial panel design	Spatial information is lost at dissociation — precisely what competition analysis requires
Deep visual proteomics	Laser microdissection of imaged cells with LC-MS/MS	Discovery-scale, unbiased	Single cell/cell class	Unbiased proteome of morphologically defined candidate winner and loser cells; can surface unanticipated fitness effectors	Low throughput; substantial input material required per class

Applications have revealed neighborhood structures that are relevant to, though not demonstrative of, competition-driven immune exclusion. In metastatic melanoma, matched CITE-seq and 40-plex tissue imaging identified an “immune-striving” microenvironment marked by peritumoral lymphoid aggregates and low intratumoral T-cell infiltration, and distinguished spatial and molecular features separating innate resistance, acquired resistance and durable response (29). Coordinated cellular neighborhoods at the colorectal cancer invasive front similarly showed that the organization of immune and stromal cells into structured spatial communities, rather than their bulk abundance, tracks with clinical outcome (34). We are explicit that immune exclusion is a description of tissue architecture: it is compatible with competitive displacement of lymphocytes, with chemokine-driven exclusion, with stromal barrier formation and with several other mechanisms, and these studies do not adjudicate between them. What they establish is the narrower and still useful point that spatial organization carries information that cell-type frequency does not.

Two practical constraints deserve emphasis. Antibody availability and specificity limit which fitness effectors can actually be measured — phospho-specific and conformational epitopes relevant to mechanical state are poorly served by existing validated panels — and segmentation errors in dense tumor tissue confound protein-level quantification precisely at the interfaces of interest (32, 33). As panels expand and image registration and segmentation pipelines mature, spatial proteomics is well positioned to become a central pillar for mapping the protein-level architecture of candidate competition networks in human tumors (36).

## 7 Artificial intelligence: foundation models as representation learners, not competition detectors

The scale and dimensionality of single-cell and spatial multi-omic data have outpaced conventional statistical pipelines, driving adoption of deep learning and, more recently, large pretrained foundation models for single-cell biology. These models are trained on tens of millions of cells and learn generalizable representations of gene expression that can be fine-tuned for cell-type annotation, batch and multi-omic integration, gene regulatory network inference and perturbation-response prediction (**Table 6**).

**Table 6:** Single-cell and spatial foundation models discussed in this review.

Model	Architecture and corpus	Modality	Defensible competition-relevant use	Reference
scGPT	Generative pretrained transformer; >33 million cells	scRNA-seq; multi-omic	Probing learned gene-gene relations to nominate regulatory programs that differ between candidate winner and loser states	Cui et al., 2024 (38)
Geneformer	Bidirectional transformer; ~30 million cells	scRNA-seq	Network inference in low-data settings, e.g. rare candidate loser populations	Theodoris et al., 2023 (39)
scFoundation	Asymmetric encoder-decoder; >50 million human cells	scRNA-seq	Read-depth-aware embeddings that reduce the risk of mistaking capture-efficiency differences between dying and healthy cells for biology	Hao et al., 2024 (40)
Nicheformer	Transformer trained jointly on dissociated and spatial data; SpatialCorpus-110M, 73 tissues	scRNA-seq + spatial transcriptomics	Imputing niche context onto existing dissociated atlases to generate candidate interface hypotheses for prospective testing	Tejada-Lapuerta et al., 2025 (41)
Novae	Self-supervised graph attention network; ~30 million cells	Spatial transcriptomics	Cross-cohort spatial domain comparison; a convenient graph substrate for the network formalism of Section 8	Blampey et al., 2025 (42)
SCimilarity	Metric-learning cell-atlas model; tens of millions of cells	scRNA-seq	Searching for a candidate loser state across published atlases, converting one observation into a pan-cancer hypothesis	Heimberg et al., 2025 (43)
GEARS	Graph neural network with gene-gene interaction priors	scRNA-seq perturbation	Prioritizing which perturbations of MYC, p53, Hippo-YAP or the ISR to test experimentally; predicted transcriptional response is not a predicted competitive outcome	Roohani et al., 2024 (44)

The final column states the competition-relevant use we consider defensible; none of these models has been validated for identifying cell competition, and all outputs are hypothesis-generating (Tier 3).

We preface this section with a caveat that applies to all of it. None of the models discussed below has been trained, benchmarked or validated for the identification of cell competition. Their published evaluations concern cell-type annotation, batch integration, domain segmentation, cell retrieval and perturbation-response prediction. Predicting a transcriptional response to a perturbation is not the same as predicting a competitive outcome between two populations, and an embedding in which two cell states are distant is not evidence that one eliminates the other. Everything in this section is therefore Tier 3 in the framework of **Table 1**: a source of prioritized hypotheses about which interfaces, states and perturbations merit experimental attention.

scGPT, a generative pretrained transformer trained on more than 33 million cells, uses self-attention over gene-gene and gene-cell relationships that can be probed to infer interaction networks and predict transcriptional responses to genetic or chemical perturbation (38). Geneformer, a bidirectional transformer trained on tens of millions of cells, was designed for transfer learning in network biology, including inference of context-specific regulatory hierarchies in low-data settings — a property that may suit rare populations that are, by construction, under-represented (39). scFoundation introduces read-depth-aware embedding, which is relevant to competition work for a specific technical reason: dying cells and healthy cells differ systematically in capture efficiency, and depth-confounded embeddings would recapitulate that artifact as biology (40).

Foundation models are now being extended into the spatial domain. Nicheformer is trained jointly on dissociated and spatially resolved data across 73 tissues and learns representations that encode the cellular niche, with the practical consequence that spatial context can be imputed onto existing dissociated atlases (41). Novae, a self-supervised graph attention network, performs zero-shot spatial domain inference with native batch correction and hierarchical domains (42); its graph formulation is convenient for the network formalism proposed in Section 8. Metric-learning atlas models such as Similarity allow a candidate loser state defined in one tumor to be searched for across published atlases (43), converting a single observation into a testable pan-cancer hypothesis. Perturbation-focused models such as GEARS incorporate gene-gene interaction priors within a graph neural network to predict outcomes of single and combinatorial perturbations (44), offering a route to prioritize which perturbations of MYC, p53, Hippo-YAP or the ISR are worth testing experimentally.

Four specific limitations temper these prospects. First, interpretability: attention weights and embedding geometry are correlational summaries of training data, not mechanistic explanations, and *post hoc* attribution methods in this setting remain poorly calibrated. Second, training-data bias: the public corpora on which these models are pretrained are dominated by a small number of tissues, technologies and, for tumor data, a small number of cancer types, so representations of rare, dying or actively eliminated populations are the least well supported part of the embedding space — precisely the part competition analysis depends upon. Third, domain shift and batch effects: performance degrades when models are applied to tissues, platforms or preservation methods outside the pretraining distribution, and reported zero-shot performance on out-of-distribution data has in several independent evaluations failed to exceed well-tuned conventional baselines. Fourth, the absence of causal validation: none of these architectures encodes an intervention, so their outputs are associations under a learned prior, and a competition network derived from a foundation model must be treated as a hypothesis requiring orthogonal functional validation before any causal claim is attached to it.

## 8 Bioinformatic inference of cell-cell communication and putative competition networks

A parallel computational thread focuses on dedicated algorithms for inferring cell-cell communication (CCC) from ligand-receptor co-expression. These provide the most direct computational proxy for the intercellular signaling that underlies both cooperative and competitive interactions in the TME (45), with the important caveat that the overwhelming majority of inferred edges reflect ordinary tissue physiology rather than competition. Their properties differ in ways that matter for competition claims (**Table 7**).

**Table 7:** Cell-cell communication inference tools, compared on the properties that determine whether a tool can support a candidate competition claim.

Tool	Scoring principle	Spatially aware	Target-gene aware	Best role in a putative competition network	Reference
Cellphone DB (v5)	Permutation test on co-expression of curated multi-subunit complexes	Yes (microenvironment prioritization)	Indirect	Captures metabolic and hormonal axes that peptide-only resources miss	Troule et al., 2025 (47); Efremova et al., 2020 (46)
CellChat	Mass-action model with network centrality and pattern recognition	Yes (v2)	No	Identifies signaling hubs; hub status is topological and is not evidence of competitive dominance	Jin et al., 2021 (48); Jin et al., 2025 (49)
NicheNet	Links ligands to observed downstream target-gene change in receiver cells	No (native)	Yes	Strongest causal proxy routinely available: nominates which signals plausibly drive stress or death programs	Browaeys et al., 2020 (50)
NATMI	Expression-weighted edge scoring; differential connectivity between conditions	No	No	Comparing network rewiring before versus after therapy	Hou et al., 2020 (51)
CytoTalk	*De novo* signal transduction network construction from integrated intra- and intercellular networks	No	Yes	Recovering a candidate intracellular relay from receptor to apoptotic effector	Hu et al., 2021 (52)
COMMOT	Collective optimal transport under spatial distance constraints	Yes (native)	Partial	Enforces the physical-proximity requirement; edges between non-adjacent populations are excluded	Cang et al., 2023 (53)
LIANA	Consensus framework wrapping multiple methods and resources	Yes (LIANA+)	Method-dependent	Guards against single-tool artifacts given documented between-tool disagreement	Dimitrov et al., 2022 (55)
Giotto	Spatial toolbox including proximity-constrained interaction enrichment	Yes	No	Neighborhood-level enrichment testing of cell-type pairs at candidate interfaces	Dries et al., 2021 (54)

All outputs are inferred edges (Tier 3) irrespective of tool.

CellPhoneDB defines a communication score from co-expression of curated multi-subunit ligand-receptor complexes (46); its fifth version substantially expanded the interaction repository, including non-peptidic ligands, extending applicability to metabolic and hormonal axes that peptide-only resources miss (47). CellChat applies a mass-action framework with network centrality and pattern recognition to identify dominant signaling sources, targets and mediators, providing a systems-level view of which populations act as communication hubs (48, 49) — hub status is a topological property and is not, by itself, evidence of competitive dominance. NicheNet takes a target-gene-centric approach, linking candidate ligands not merely to receptor expression but to downstream transcriptional change in receiving cells (50); this is the strongest causal proxy routinely available, because it can nominate which winner-derived signals plausibly drive stress or death programs in a neighboring population rather than merely co-occurring with them, although the inferred link remains associational. NATMI emphasizes differential connectivity between conditions (51), useful for comparing network rewiring before and after therapy, and CytoTalk constructs *de novo* intracellular signal transduction networks (52), which is how one might recover a relay from receptor engagement to apoptotic effector within a candidate loser cell.

Two developments are especially pertinent. First, spatial constraint: COMMOT formulates communication as collective optimal transport under spatial distance constraints (53), and neighborhood-enrichment testing within spatial toolboxes (54) enforces the physical-proximity requirement that competition demands. An inferred winner-to-loser edge between two populations that never touch is not a candidate competitive interaction at all. Second, consensus: LIANA wraps multiple methods and resources into a harmonized framework (55), guarding against single-tool artifacts given documented between-tool disagreement.

### 8.1 A falsifiable definition of a putative cell competition network

Together these frameworks allow a putative cell competition network to be defined formally rather than metaphorically. Let the node set comprise cell states typed as candidate winner, candidate loser or bystander, each assignment supported by explicit transcriptional, proteomic and morphological evidence, and each annotated with the evidence tier (**Table 1**) on which it rests. Let the edge set comprise four distinguishable relations: (i) inferred ligand-receptor signaling; (ii) spatial adjacency, expressed as shared interface length; (iii) metabolic competition for a shared limiting resource; and (iv) mechanical or contact-dependent interaction, represented by interface curvature, cell compaction or density gradients, junctional and actomyosin marker asymmetry, and extrusion morphology. The inclusion of a mechanical edge type is not optional. Mechanical competition is one of the three principal modes of cell competition (**Table 3**), it is the dominant mode in several of the best-characterized mammalian systems, and a framework that omits it is incomplete by construction and will systematically mis-assign competitive mode in stiff or crowded tumor regions.

Each edge should carry three attributes: a weight reflecting the multi-omic evidence supporting it; a direction from candidate winner to candidate loser, where directionality can be assigned; and, critically, an evidence tier. We distinguish two edge classes. An inferred edge is a statistical or model-derived relationship (Tier 3) or a measured spatial or molecular association (Tier 2). A demonstrated edge is one for which an elimination, extrusion, forced-differentiation or displacement event has been directly observed and shown to depend on the presence of the putative winner (Tier 1). In current human tumor datasets, essentially all edges are inferred edges; demonstrated edges exist almost exclusively in experimental systems. Networks should be reported with this annotation visible, so that a reader can see at a glance how much of a given topology rests on measurement and how much on inference.

Stating the object this precisely matters because it makes the framework falsifiable: a competition-dominant topology (directed, asymmetric, interface-localized) is distinguishable from a cooperation-dominant one (reciprocal, distributed), and the distinction can be tested rather than asserted.

### 8.2 Minimum evidence required to validate a predicted winner-loser interaction

Because the value of an inferred network lies entirely in what it prioritizes for testing, it is worth stating concretely what would be required to convert a predicted winner-loser edge into a demonstrated one. We suggest five elements. First, an independent demonstration that the two populations are in direct contact or within the effective range of the proposed mediator, established at single-cell segmentation resolution and supported by serial sectioning to exclude a sectioning artifact. Second, evidence of an outcome in the candidate loser population at the interface — cleaved caspase-3, extrusion morphology, forced differentiation markers or a measured reduction in clone representation over time — quantified as a function of distance from the interface rather than across the section as a whole. Third, a context-dependence control: the candidate loser population must be shown to be viable in the absence of the candidate winner, in a matched model system, since this is what separates competition from intrinsic fragility. Fourth, a perturbation of the proposed mediator — ligand or receptor blockade, transporter inhibition, or mechanical modulation such as substrate stiffness or ROCK inhibition for mechanical edges — that abolishes or reverses the outcome. Fifth, where feasible, longitudinal or treatment-paired sampling in the human setting to confirm that the predicted direction corresponds to an actual change in clone representation over time. A predicted edge that has satisfied elements one to four in a matched model, and element five in the originating human cohort, may reasonably be described as a demonstrated competitive interaction; anything less should retain the “putative” qualifier.

## 9 Toward integrative multi-omic analysis and a conceptual Cell Competition Score

No single technology captures the multi-scale nature of cell competition. Genomic and transcriptomic data indicate which clones exist and what programs they run; spatial transcriptomics and proteomics indicate where those clones sit relative to their neighbors and what functional state they are in; AI-based network inference connects these layers into a predictive model. Multi-omic integration is what raises an observation from a co-occurrence of states to a spatially and molecularly specified candidate interaction. It does not, on its own, convert inference into demonstration. **Figure 3** summarizes this progression from spatial heterogeneity through network inference to potential therapeutic application, and we stress that panels B and C of that figure depict a proposed framework rather than validated practice.

**Figure 3:**
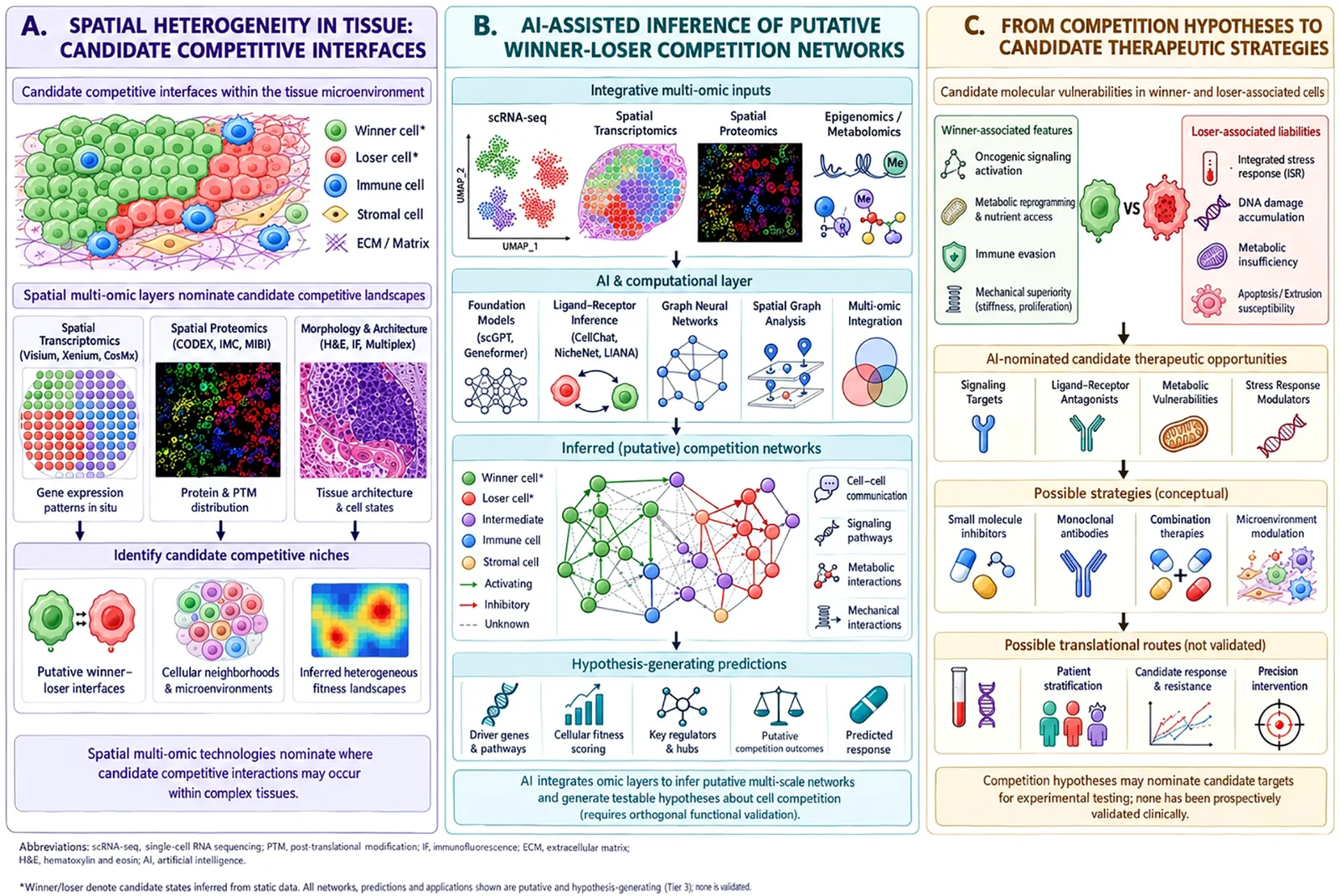
From spatial heterogeneity to inferred competition networks and potential therapeutic application: a proposed framework. **(A)** Spatial heterogeneity in tissue: spatial transcriptomics, spatial proteomics and tissue morphology and architecture nominate candidate competitive interfaces and niches within the tumor microenvironment. **(B)** AI-assisted inference of putative winner–loser competition networks: multi-omic inputs are combined with foundation models, ligand–receptor inference, graph neural networks and spatial graph analysis to infer putative competition networks and hypothesis-generating predictions. **(C)** From competition hypotheses to candidate therapeutic strategies: winner-associated features and loser-associated liabilities may suggest conceptual therapeutic strategies and translational routes. Winner and loser denote candidate cell states inferred from static data; all networks, predictions and applications shown are putative and hypothesis-generating, and none has been validated.

Emerging integrative studies illustrate the value of combining modalities. Matched CITE-seq and 40-plex tissue imaging in melanoma allowed transcriptomic subclone identification to be overlaid on spatial protein architecture, revealing resistance-associated cellular communities that neither modality alone could resolve (29). Spatial foundation models make a reverse operation feasible: spatial context learned from imaging-based data can be imputed onto the large existing corpus of dissociated tumor atlases (41), which means competition-oriented reanalysis need not wait for new spatial cohorts in every tumor type — though imputed context is itself a prediction and inherits every caveat of Section 7.

Extending this logic, we outline a conceptual composite Cell Competition Score (CCS) integrating five measurement layers: transcriptional fitness, stress and death priming, spatial adjacency, protein-level effector state, and proximity-constrained inferred signaling (**Table 8**). We wish to be unambiguous about its status. The CCS is a specification for an experiment, not a biomarker and not a validated instrument. No component has been prospectively tested, no weighting function has been fitted, no training or independent validation cohort exists, and the score has not been benchmarked in any cancer type. It should not be described as biomarker-ready, and we do not propose it for any clinical use.

**Table 8:** Conceptual specification for a context-dependent Cell Competition Score (CCS).

Layer	Measurable feature	Assay	Expected direction (context-specific; must be established locally)	Normalization	Validation required before use
Transcriptional fitness	MYC target activity; p53 pathway output; YAP/TEAD target signature	scRNA-seq or spatial transcriptomics, scored per cell	Direction is mode-dependent: p53 output marks losers in the checkpoint mode but winners are p53-low only in some settings, and high p53 marks losers in mechanical competition; YAP/TEAD marks winners in some tissues and the outcompeting normal compartment in others. No universal direction is assumed.	Relative to matched adjacent normal epithelium in the same section	Perturbation of the pathway in a matched model, establishing the local direction
Stress and death priming	ISR activation (ATF4 targets); apoptotic priming; extrusion markers	Spatial transcriptomics plus cleaved-caspase imaging	Elevated in candidate losers in most described settings; note that ISR activation is cytoprotective in ref. 12, so elevation alone is ambiguous	Per-neighborhood z-score rather than per-section mean	Live-imaging or organoid extrusion assay demonstrating outcome
Spatial adjacency	Fraction of a clone perimeter in contact with a fitness-divergent population	Single-cell-resolution spatial transcriptomics or multiplexed imaging	A non-trivial shared interface is necessary but not sufficient for competition	Interface length normalized to clone perimeter	Serial sectioning to exclude sectioning artifact; segmentation quality metrics reported
Protein-level effector state	Cortical tension proxies; transporter abundance; caspase activation	Imaging mass cytometry, MIBI-TOF or CODEX	Directional asymmetry across the interface; sign established locally	Interface-anchored distance binning	Traction force microscopy or stiffness mapping
Mechanical/contact interaction	Interface curvature; local density and compaction gradient; junctional and actomyosin asymmetry	Multiplexed imaging with morphometric analysis; matched biomechanical measurement where available	Compaction and compressibility asymmetry expected towards the candidate loser in mechanical-mode competition	Distance-binned relative to the interface, within neighborhood	Substrate stiffness or ROCK modulation in a matched model
Inferred signaling	Proximity-constrained ligand-receptor edges between the two populations	Consensus inference restricted to short-range pairs	Directed edge weight from candidate winner to candidate loser exceeds the reverse	Permutation null preserving spatial autocorrelation	Ligand blockade in a matched model
Composite CCS	Weighted combination of the layers above	Integrated pipeline	Continuous score reported per neighborhood; interpreted as competitive potential, not as demonstrated competition	Weights fitted on a designated training cohort and reported explicitly	Independent cohort on a different platform, then prospective pre-registered evaluation

This is a proposed, testable framework, not a validated instrument and not a biomarker. Expected directions must be established by Tier 1 evidence in a matched model for each tissue and competitive mode before the score is computed; they are not transportable across contexts.

A second limitation is more fundamental than the absence of validation data, and follows directly from Section 3. The expected directions of the transcriptional fitness layer cannot be fixed *a priori* across tumor types. MYC dosage predicts winner status in the settings of references 7 and 19, but the p53 term reverses sign between the tumor-suppressor-checkpoint mode (20) and the mechanical mode (16), and YAP/TEAD activity marks winners in one system (22) and is associated with the outcompeting normal compartment in another (21). A single CCS with universal winner and loser directions across cancers is therefore not currently defensible, and we do not propose one. What we propose instead is a family of context-specific instantiations: within a defined tissue, competitive mode and clonal comparison, the directions of each layer are first established by Tier 1 evidence in a matched model, and only then is the score computed and its weights fitted. The score also measures molecular states consistent with competitive potential; it does not measure whether competition has occurred, and a high CCS in a fixed section is a hypothesis about that tissue, not a finding.

Accordingly, the validation pathway matters more than the score. Any instantiation would require, in sequence: (i) context-specific establishment of layer directions by perturbation in a matched experimental model; (ii) explicit specification and public reporting of the weighting function, fitted on a designated training cohort; (iii) evaluation in at least one independent cohort profiled on a different platform, to expose batch and domain-shift effects; (iv) perturbation-based validation that manipulating the highest-weighted layer changes the competitive outcome; and (v) prospective, pre-registered assessment against a clinical endpoint before any association with outcome is described as predictive. Steps (i) to (iv) have not been completed for any of the five layers, and step (v) is several stages beyond current capability.

## 10 Potential implications for precision oncology

Reframing tumor heterogeneity through the lens of cell competition suggests several directions, all of which remain hypothesis-generating. We state at the outset that no cell-competition-derived measurement is currently ready for clinical use, that no competition network analysis has been prospectively validated against a clinical endpoint, and that the arguments below describe candidate strategies requiring substantial preclinical and clinical work.

First, because super-competitor clones may actively eliminate both normal tissue and less-fit neoplastic subclones, therapies that shift competitive balance rather than only killing malignant cells outright represent an underexplored strategy. This is supported by direct experimental evidence: dietary or genetic reprogramming of tumor-associated macrophages was sufficient to convert them into winners against MYC-overexpressing cancer cells and to slow tumor growth *in vivo* (13), and peritumoral activation of YAP and TAZ enabled normal hepatocytes to outcompete transformed cells and suppress liver cancer (21). Both are preclinical demonstrations in mouse models; both nonetheless establish that the competitive threshold is a manipulable variable, and both suggest that a relevant therapeutic target may sit in the non-malignant compartment.

Second, spatial characterization of the immune microenvironment is informing candidate biomarkers of immunotherapy response, though not yet on competition-specific grounds. Structured immune-stromal neighborhoods track with outcome more faithfully than bulk immune cell frequency (34), and longitudinal multimodal profiling in melanoma distinguished innate from acquired resistance by spatial and molecular organization rather than cell-type abundance alone (29). Whether any part of that signal reflects competitive displacement as opposed to chemokine-driven exclusion or stromal barrier formation is untested. The defensible implication for companion diagnostics is narrower than a competition framing would suggest: future assays may need to incorporate spatial architecture rather than relying solely on single-marker immunohistochemistry, and whether a specifically competition-derived feature adds information beyond existing spatial features is an open, testable question.

Third, competition offers a candidate framework for early detection and risk stratification. Morphologically normal human epithelium is extensively colonized by mutant clones (27), and Apc-mutant supercompetitors initiate intestinal tumors by biasing clonal competition in their own favor (23, 24). Identifying competitively dominant clones before full malignant transformation is a coherent research objective for surveillance in high-risk tissues, and one for which spatial assays are in principle better suited than bulk sequencing, since the relevant signal is the geometry of clonal boundaries rather than mutation burden alone. No such assay has been developed or evaluated.

Fourth, and most speculatively, loser-cell vulnerabilities may constitute a distinct target class. Cells under competitive stress depend on the ISR for survival in the model system of reference 12, raising the possibility of therapeutically converting a surviving loser population into an eliminated one; this has not been tested in a tumor setting. The risks cut both ways: interventions that shift competitive balance could inadvertently select for the most competitive clone, and it has been proposed, on preprint evidence, that competitively displaced cells may be reprogramed towards dissemination rather than death (10, 28). Any therapeutic strategy built on modulating competition must therefore be evaluated for the phenotype of the cells it displaces, not only for the bulk response it produces. More broadly, tumor-associated signaling dependencies such as VEGFR2-driven angiogenic signaling are being explored as therapeutic targets in cancer (59), although such studies do not address competition itself.

## 11 Challenges and limitations

Substantial obstacles stand between current capability and any clinical translation. The most consequential is that inference is not causation. Current CCC and foundation-model approaches infer association from co-expression, spatial proximity or learned priors; they do not establish that a given interaction causally drives loser-cell elimination. Few published studies pair computational inference with functional perturbation *in vivo*, and until that becomes routine, an inferred competition network should be read as a prioritized hypothesis set and nothing more.

Benchmarking reinforces this caution. Systematic evaluation integrating scRNA-seq with spatial information found that inferred interactions separate into short- and long-range classes by ligand-receptor spatial distance, and that agreement between methods on commonly inferred interactions was unstable across tissues (56) — implying that only short-range, spatially constrained predictions should be advanced as competition candidates. Comparison across methods and resources showed that the choice of underlying resource influences results as much as the choice of algorithm (55), so the resource must be reported, not only the tool. Systematic framework-level evaluation found that no single method dominates across task types and that integration outperforms individual tools (57), supporting consensus rather than single-tool networks. Method rankings are benchmark-dependent, and a tool leading on one benchmark can rank near the bottom on another; rankings therefore cannot be quoted context-free (45). **Table 9** summarizes this evidence. We note additionally that none of these benchmarks evaluates the task of interest here — no benchmark exists for the recovery of competitive interactions — so the reliability figures quoted are upper bounds derived from an easier problem.

**Table 9:** Benchmarking evidence bearing on the reliability of inferred cell-cell communication, and by extension of putative competition networks.

Benchmark	Scope	Principal finding	Consequence for competition inference	Reference
Integration of scRNA-seq with spatial information	16 cell-cell interaction methods evaluated against spatial data	Interactions separate into short- and long-range classes by ligand-receptor spatial distance; agreement on commonly inferred interactions was unstable across tissues	Only short-range, spatially constrained predictions should be advanced as competition candidates	Liu et al., 2022 (56)
Comparison of methods and resources (LIANA)	Systematic comparison of methods and of the underlying ligand-receptor resources	Choice of resource influences results as much as choice of method; top-ranked interactions vary substantially between tools	Report the resource, not only the tool; consider consensus ranking	Dimitrov et al., 2022 (55)
ESICCC framework	Framework for evaluation, selection and integration of CCC methods	No single method dominates across task types; integration outperforms individual tools	Supports a consensus rather than single-tool network	Luo et al., 2023 (57)
Review of cell-cell interaction methodology	Evolution of cell-cell interaction methods and their benchmarks	Rankings are benchmark-dependent; next-generation tools increasingly incorporate spatial organization, multiple ligand classes and intracellular signaling	Rankings cannot be quoted context-free; state which benchmark supports a claim	Armingol et al., 2024 (45)

No benchmark currently evaluates recovery of competitive interactions specifically.

Technical constraints compound the statistical ones. Spatial platforms involve inherent resolution-throughput trade-offs: array-based methods sacrifice single-cell resolution, while imaging-based platforms are constrained by panel size, tissue coverage and cost. Targeted panels must be pre-specified, which means fitness markers must be hypothesized in advance — a real limitation when the goal is discovering unanticipated fitness effectors. Segmentation errors in dense tumor nests are a particular hazard because they manufacture exactly the artifact under investigation (32, 33).

There are also conceptual difficulties specific to competition, and these are the deepest. Winner and loser status is context-dependent rather than intrinsic: YAP activity is associated with winner status in some settings and with the outcompeting normal compartment in others (21, 22), and the sign of the p53 term reverses between competitive modes (16, 20), so signature-based assignment cannot be transported between contexts without revalidation. Distinguishing genuine competitive elimination from immune editing, drug-induced transcriptional plasticity or simple differential proliferation (**Table 2**) requires longitudinal, treatment-paired sampling that remains scarce. Fixed human tissue provides a single time point, and competition is defined by change over time; this is a limitation of the material rather than of any platform, and no increase in molecular resolution overcomes it. Existing atlases are biased towards a small number of well-studied tumor types, and truly pan-cancer, longitudinally sampled, treatment-paired datasets are rare. Finally, two claims discussed in this review rest on preprints (18, 28); these are labeled explicitly, the surrounding language is hedged, and neither is load-bearing for the argument of the review.

## 12 Future directions

Several directions appear particularly promising. First, spatial multi-omic foundation models should be trained jointly across transcriptomic, proteomic and histopathological modalities, so that candidate winner and loser states become comparable across cancer types and technologies within a shared embedding space (41, 42), with explicit evaluation of domain shift and batch effect rather than reliance on reported zero-shot performance. The immediate practical gain is the ability to search for a competitively defined cell state across published atlases rather than within one dataset (43).

Second, perturbation-prediction models should be coupled to experimental validation in a closed loop. In silico simulation of how targeting MYC, p53, Hippo-YAP or the ISR shifts competitive balance (44) is useful only if the predictions are tested; the value of these models lies in prioritizing which of a large number of candidate perturbations is worth performing, not in replacing the experiment.

Third, longitudinal and treatment-paired sampling — combining scRNA-seq, spatial transcriptomics, spatial proteomics and repertoire sequencing from the same patients before and during therapy — is essential for distinguishing genuine competitive elimination from the confounding processes in **Table 2**. This is the single most valuable and least available resource in the field, and study designs of the kind demonstrated in melanoma (29) provide a workable template.

Fourth, benchmarking consortia analogous to those established for CCC inference (55, 57) should be extended to spatial foundation models and to competition-network inference specifically, with community standards for validating inferred competitive interactions against orthogonal functional assays. A benchmark in which methods are evaluated on their ability to recover known competitive interactions in genetically defined mosaic systems, where ground truth exists, would be the most useful single contribution the computational community could make to this problem. Standardized, quantitative image-analysis pipelines for competition experiments already exist in model systems (58) and provide a precedent.

Finally, mechanical and metabolic cues are central to competition but are poorly captured by any sequencing-based platform. Integration of biomechanical measurement — traction force microscopy, stiffness mapping — and metabolomics imaging with spatial multi-omic atlases represents the most important gap between what current technology measures and what the biology requires (11, 18).

## 13 Conclusion

Cell competition, once studied primarily in developmental model organisms, is now recognized as a potentially important and possibly targetable process in human cancer, with implications for clonal evolution, immune escape and metastatic dissemination. The convergence of single-cell RNA sequencing, spatial transcriptomics, spatial and single-cell proteomics, artificial intelligence and network-inference bioinformatics provides the technical means to measure, at the required resolution, the cellular states and tissue configurations in which competition could occur, and to nominate specific candidate winner-loser interactions for testing. It does not yet provide the means to demonstrate competition in human tissue, and we have argued throughout that the distinction between a microenvironment consistent with competition and a demonstrated competitive interaction should be maintained explicitly rather than elided.

Realizing the potential of this convergence will require sustained investment in multi-omic integration standards, in causal and functional validation of computationally inferred interactions, in benchmarks with genuine ground truth, and eventually in prospective clinical studies. The demonstrations that competitive balance can be shifted therapeutically *in vivo* indicate that the underlying premise is sound; what remains is the considerable work of making it measurable, reproducible and — only then — clinically actionable.
